# Supplementary material for: Variability in an effector gene promoter of a necrotrophic fungal pathogen dictates epistasis and effector-triggered susceptibility in wheat
Source: PLoS Pathog. 2022 Jan 6;18(1):e1010149. doi: 10.1371/journal.ppat.1010149 (PMC8735624; doi:10.1371/journal.ppat.1010149)

>401 bp element

ACGCTGTACGCTATACGCAGACTGTTAGGAGATCACACCTTATATATGCTGTTTTACTCCTTTACTCTATAGTCCTATCAATCGTGTACTTAGTCGAGAGTTCTAGTATATTAGGTTCGCAACTTACATTAGGAAGGCGACTAATCATACTGTAAGGTGAAATTGGCAGAAGCCTTCAGGAAAATCTCTAGATACCTATGGTAGCTCTGAAGGAAGCTTACAGCCGTTTGCTGACAGGTTCGAGCACATACTACAGACATCTAATCTTAGCCAATATTCTGCCCCTGCGTCAGCATTAAGGACTTCCAATTAATTAGTCTGTTCTAGGAAACCTCAGAGCTTCCCGCAAATCCAAGGAATATAGTACAACAGCAGAGAGGCCTCTGAGACCTATTCTTAAT

>401 bp spacer

GCTTGCTCCTTTCGCTTTCTTCCCTTCCTTTCTCGCCACGTTCGCCGGCTTTCCCCGTCAAGCTCTAAATCGGGGGCTCCCTTTAGGGTTCCGATTTAGTGCTTTACGGCACCTCGACCCCAAAAAACTTGATTAGGGTGATGGTTCACGTAGTGGGCCATCGCCCTGATAGACGGTTTTTCGCCCTTTGACGTTGGAGTCCACGTTCTTTAATAGTGGACTCTTGTTCCAAACTGGAACAACACTCAACCCTATCTCGGTCTATTCTTTTGATTTATAAGGGATTTTGCCGATTTCGGCCTATTGGTTAAAAAATGAGCTGATTTAACAAAAATTTAACGCGAATTTTAACAAAATATTAACGTTTACAATTTCAGGTGGCACTTTTCGGGGAAATAATG


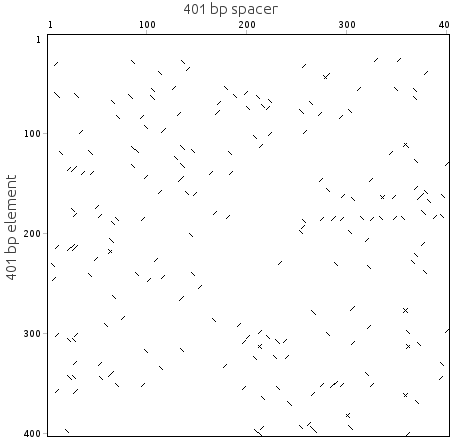

Supplement: S2 File — A dotplot is provided to demonstrate the sequence dissimilarity. (DOCX) [file ppat.1010149.s002.docx]
