# Supplementary figures and images for: Variability in an effector gene promoter of a necrotrophic fungal pathogen dictates epistasis and effector-triggered susceptibility in wheat
Source: PLoS Pathog. 2022 Jan 6;18(1):e1010149. doi: 10.1371/journal.ppat.1010149 (PMC8735624; doi:10.1371/journal.ppat.1010149)

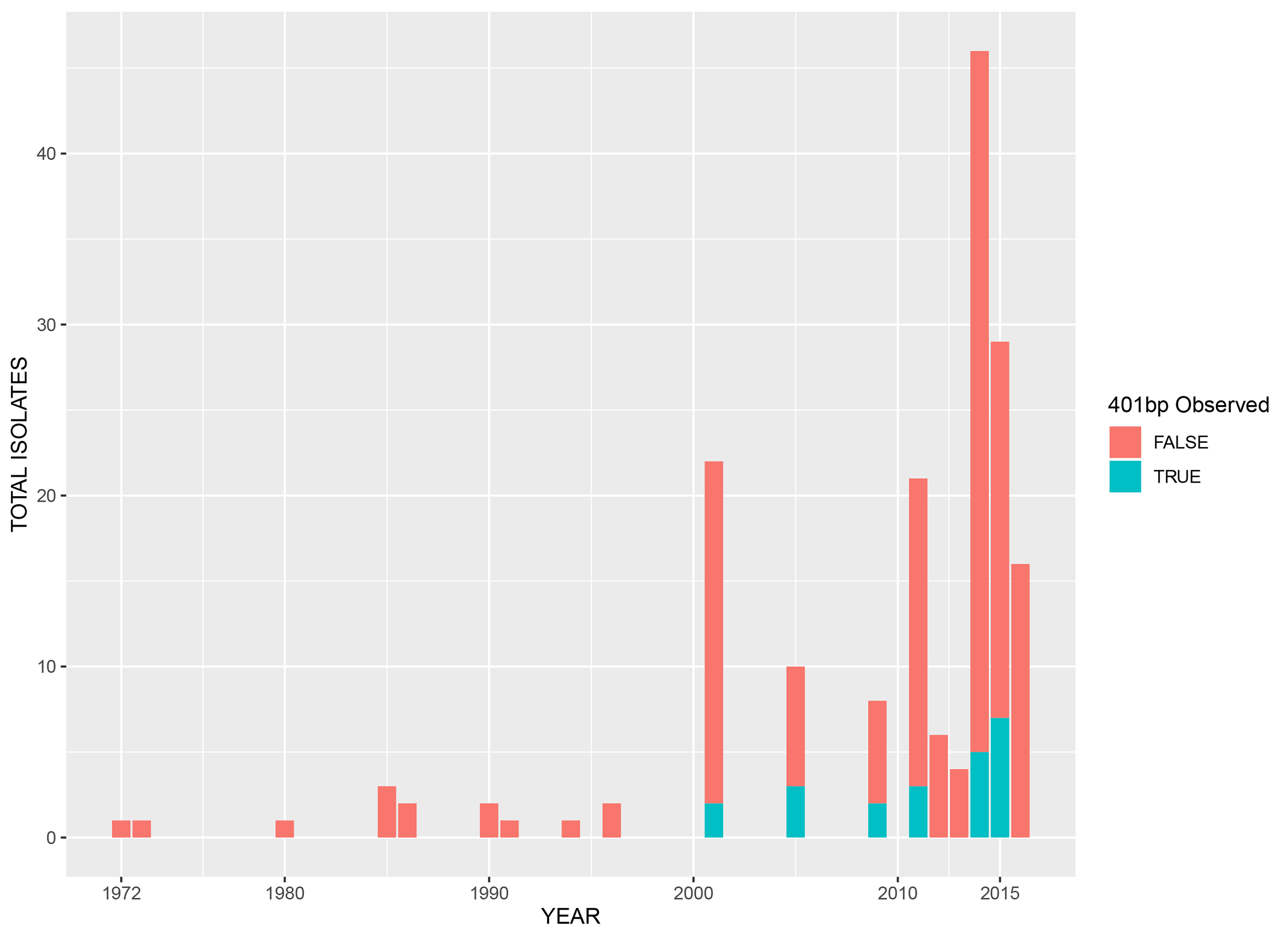

Supplement: S1 Fig — “TRUE” indicates the presence of PE401 whereas “FALSE” indicates the absence of PE401. (JPG) [file ppat.1010149.s004.jpg]

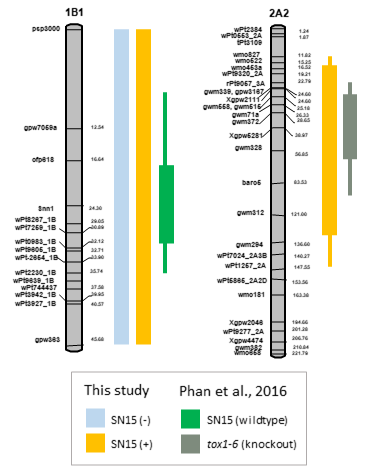

Supplement: S2 Fig — (PNG) [file ppat.1010149.s005.png]

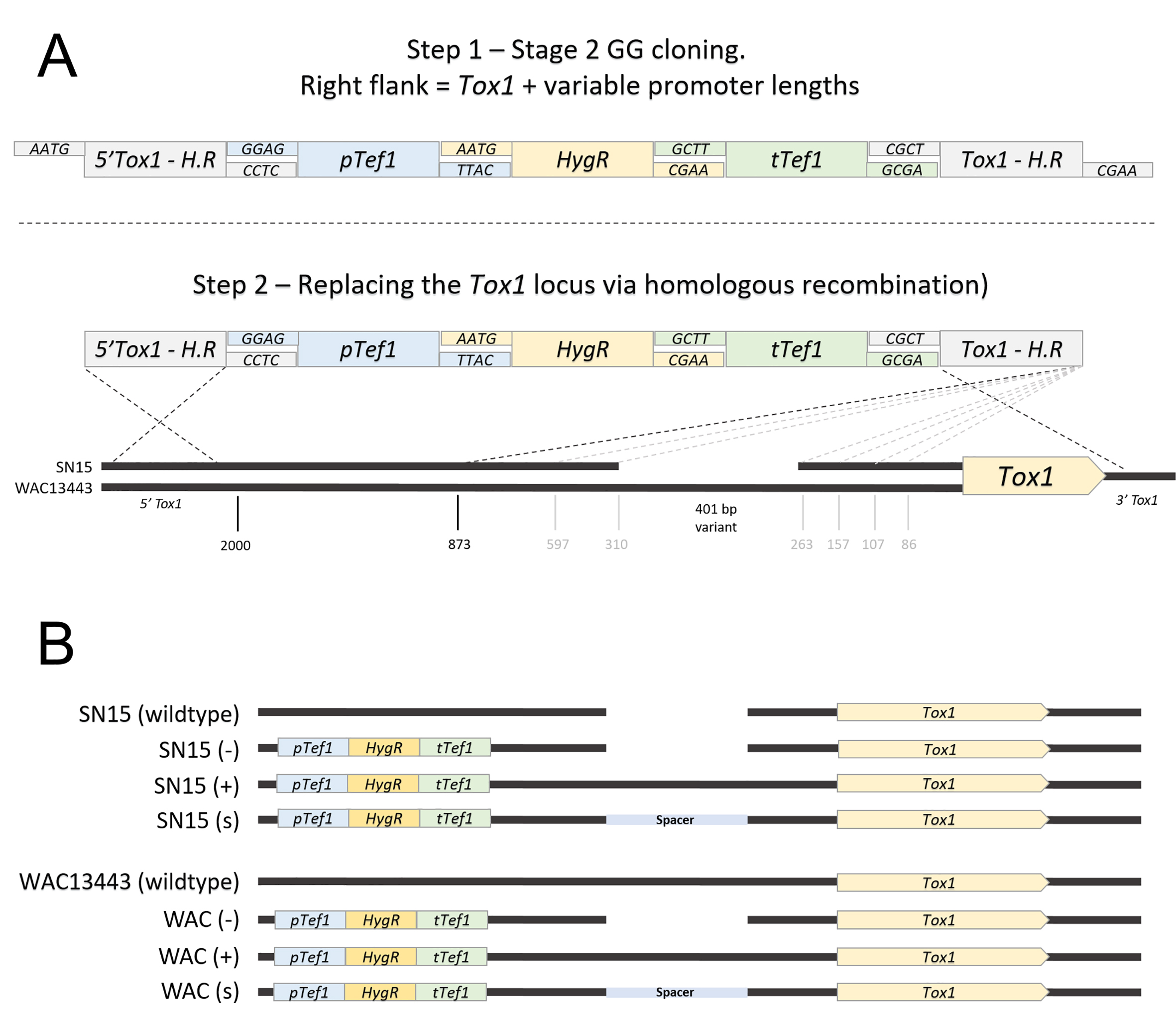

Supplement: S3 Fig — A. In step 1, Golden Gate cloning was used to assemble constructs for promoter replacement at the Tox1 locus. Linear replacement constructs were amplified for fungal transformation in step 2. B. Promoter replacement mutants generated in the study representing the Tox1 locus in the respective background strains. (JPG) [file ppat.1010149.s006.jpg]
